# Diversity and Functions of Type II Topoisomerases

**DOI:** 10.32607/actanaturae.11058

**Published:** 2021

**Authors:** D. A. Sutormin, A. K. Galivondzhyan, A. V. Polkhovskiy, S. O. Kamalyan, K. V. Severinov, S. A. Dubiley

**Affiliations:** Institute of Gene Biology RAS, Moscow, 119334 Russia; Centre for Life Sciences, Skolkovo Institute of Science and Technology, Moscow, 121205 Russia; Lomonosov Moscow State University, Moscow, 119991 Russia; Institute of Molecular Genetics RAS, Moscow, 123182 Russia; Centre for Precision Genome Editing and Genetic Technologies for Biomedicine, Institute of Gene Biology RAS, Moscow, 119334 Russia; Waksman Institute for Microbiology, Piscataway, New Jersey, 08854 USA

**Keywords:** topoisomerases, supercoiling, decatenation, transcription, replication, DNA segregation, spatial chromosome organization

## Abstract

The DNA double helix provides a simple and elegant way to store and copy genetic information. However, the processes requiring the DNA helix strands separation, such as transcription and replication, induce a topological side-effect – supercoiling of the molecule. Topoisomerases comprise a specific group of enzymes that disentangle the topological challenges associated with DNA supercoiling. They relax DNA supercoils and resolve catenanes and knots. Here, we review the catalytic cycles, evolution, diversity, and functional roles of type II topoisomerases in organisms from all domains of life, as well as viruses and other mobile genetic elements.

## DNA TOPOLOGY

**Fig. 1:**
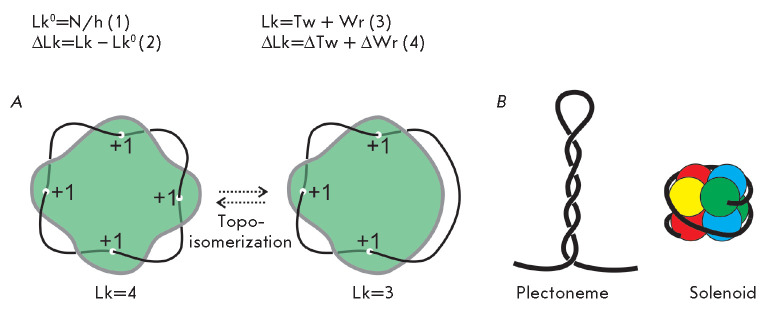
DNA topology. (*A*) Linking number of a circular DNA molecule and changes in the linking number resulting from strand cleavage and transfer. (*B*) Spatial structures, plectoneme and solenoid, arising from DNA supercoiling

The topological state of DNA and the level of its supercoiling are described using the linking number concept (Lk) [1]. If one of the strands of a covalently closed circular DNA molecule is thought to be the edge of an imaginary surface, then the linking number of DNA strands is the number of intersections of this surface with the second DNA strand, with allowance for the sign of this intersection (*Fig. 1A*). Lk does not depend on molecule deformations and can only be altered through cleavage, passage, and religation of DNA strands (*Fig. 1A*) [2]. For a relaxed DNA molecule, the theoretical linking number (Lk0) can be calculated as a ratio between the DNA length in base pairs (N) and the period of DNA (h = 10.5 bp/turn for the canonical B-form of DNA) (1). Lk of DNA molecules isolated from living organisms differs from Lk0: it can either exceed Lk0 (ΔLk > 0, a positively supercoiled molecule) or be less than Lk0 (ΔLk < 0, a negatively supercoiled molecules) (2). Lk is the sum of two geometrical parameters of the double helix, called the twist (Tw) and the writhe (Wr) (3). The twist is defined as the number of times DNA chains turn around each other along the double helix axis, while the writhe is a measure of the supercoiling of the DNA axis [3]. When Lk is different from Lk0, supercoiling is partitioned between the twist and writhe (4), which can interconvert to each other. For example, according to the electron microscopy of plasmids, the writhe and twist account for 75% and 25% of DNA supercoiling, respectively [3]. In nature, supercoiled DNA in the form of writhe stably exists in two forms: plectoneme (a higher order double helix) and a solenoid (a higher order single helix, which is typical of DNA wrapped around a protein) (*Fig. 1B*) [3]. A more detailed and comprehensive discussion of DNA topology may be found, for example, in the book *DNA Topology *by Bates & Maxwell, 2005 [3].

## STRUCTURE, EVOLUTION, AND CATALYTIC MECHANISM OF TYPE II TOPOISOMERASES

Special enzymes, topoisomerases, regulate the level of DNA supercoiling and resolve knots and catenanes [4, 5]. According to their structure, homology, and catalytic mechanism, topoisomerases are usually divided into type I and type II [4]. Type I topoisomerases introduce a single-strand DNA break (nick) and alter the supercoil ing state of a molecule either by rotating the DNA duplex around the intact second strand (class IB, change Lk of the molecule by an arbitrary integer number per catalytic event) or by passing the intact strand through the nick (class IA, change Lk by ±1 per catalytic event). Type II topoisomerases cleave both strands in a DNA fragment, termed the G-segment, and pass the second duplex, the T-segment, through this break, hydrolyzing two ATP molecules (*Fig. 3*) [6, 7, 8]. This process is topologically equivalent to a change in Lk by ±2 [9]. DNA supercoiling is altered if G- and T-segments belong to the same molecule, but if they come from different molecules, action of the toposiomerase results in catenation or decatenation of DNAs (*Fig. 3C*). Below, we will analyze the diversity, mechanisms, and physiological role of type II enzymes.

Type II topoisomerases are found in organisms of all domains of life and are encoded in most, except for a few extremely reduced ones, sequenced genomes of cellular organisms [10, 11]. In all studied cases, type II topoisomerases have been shown to be necessary for transcription, replication, and segregation of chromosomes during cell division.

**Fig. 2:**
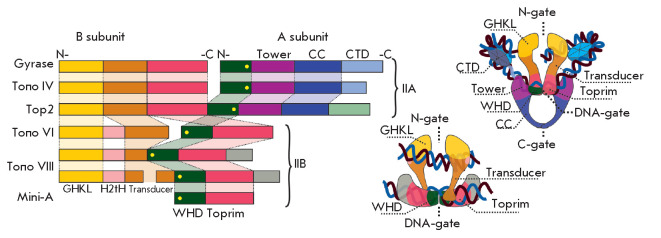
Type II topoisomerase structure. *Left *– variants of the enzyme domain architecture. Homologous domains are shown in the same colors. In the WHD, the catalytic tyrosine residue responsible for DNA cleavage is depicted by a yellow circle. *Right *– domain organization of type IIA (DNA gyrase) and IIB (Topo VI) topoisomerases

**Fig. 3:**
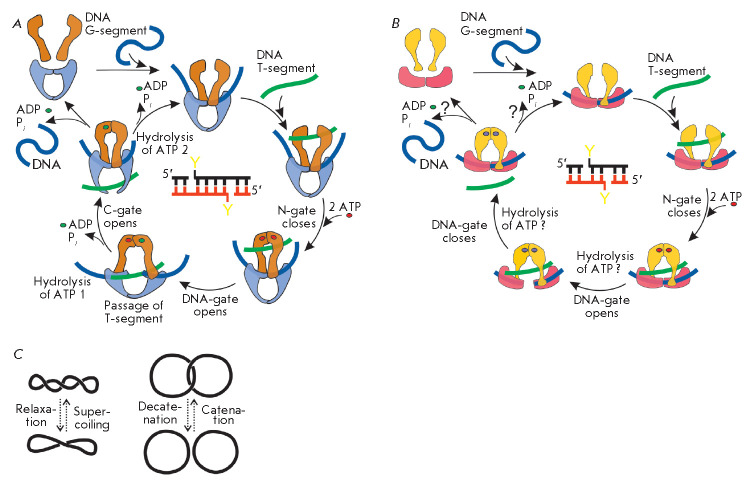
Catalytic cycles of the topoisomerases IIA (*A*) and IIB (*B*) and the effect of topoisomerase activity on DNA topology (*C*). The scheme shows the following steps: binding of the DNA G-segment (blue) and T-segment (green); binding and hydrolysis of ATP molecules (ATP – red circle, ADP – green circle, if the bound nucleotide state is unknown (ATP/ADP), it is depicted by a purple circle); cleavage and ligation of the G-segment and passage of the T-segment through the enzymatic complex. A scheme for G-segment cleavage is shown in the center of each cycle (Y – catalytic tyrosine residue of the WHD). Type II topoisomerases are able to change DNA supercoiling, as well as unlink (decatenate) or link (catenate) DNA molecules

On the basis of their structure and catalytic cycle features, type II topoisomerases are subdivided into two classes: IIA and IIB (*Fig. 2*, *Fig. 3*) [4]. Topoisomerases can be either heterotetramers consisting of two B and two A subunits or homodimers in which the B and A subunits are combined into a single polypeptide [10]. The topoisomerase subunits have dimerization interfaces, referred to as gates. The conserved ATP-hydrolysis GHKL (**G**yrase, **H**sp90, **H**istidine Kinase,** M**utL) domain [12] forms the **N-gate**, and the Toprim and WHD (**Topo**isomerase/Primase and **W**inged-**h**elix** d**omain) domains form the **DNA-gate **[13]. The G-segment of DNA binds to the DNA-gate region of the enzyme and is cleaved by active site tyrosyl residues of the WHD domains [14]. The third dimerization interface (**C-gate**), formed by the **c**oiled-**c**oil (CC) domain, is present only in type IIA enzymes (*Fig. 2*) [15]. The **C**-**t**erminal **d**omains (CTD) are located either at the C-termini of A-subunits or at the end of fused polypeptides. CTD determines the specificity of topoisomerases IIA to DNA structures (supercoils or crossovers), interacts with other proteins, and, in eukaryotes, is subject to post-translational modifications regulating the activity of the enzyme [16, 17, 18].

At the first stage of the catalytic cycle, topoisomerase IIA is believed to bind the G-segment of DNA in the DNA-gate region [19]. The binding causes DNA bending, which is probably the basis of the topological scanning of DNA by the enzyme: topoisomerase preferentially binds to supercoiled regions of the molecules that are either already bent or can be easily bent due to energy of supercoiling [20, 21, 22]. Next, the T-segment of DNA is trapped between the GHKL domains and the DNA-gate. Binding of two ATP molecules to ATPase centers leads to dimerization of the GHKL domains, closure of the N-gate, and secure capture of the T-segment [23]. Hydrolysis of the first ATP molecule to ADP triggers cleavage of the DNA G-segment by the catalytic site tyrosyl residues of the WHDs and opens the DNA-gate, which results in the T-segment passage through the break to the protein cavity at the C-gate [7, 13, 24, 25]. To stabilize the double-stranded break, the hydroxyl groups of the tyrosyl residues remain linked to the DNA 5’-ends by phosphodiester bonds. Opening of the C-gate, which releases the T-segment from the enzymatic complex, follows closure of the DNA-gate and ligation of the G-segment due to hydrolysis of the second ATP molecule [26]. The release of ADP molecules, which have low affinity for active centers, leads to the opening of the N-gate and transition of the enzyme to its original state (*Fig. 3A*) [23].

Binding of ATP molecules is believed to be necessary for the unidirectional passage of the T-segment, since this segment is incapable of leaving the enzyme through the N-gate until both ATP molecules are hydrolyzed [24]. It should be noted that the role of ATP hydrolysis in segment passage has not been fully elucidated. According to one of the existing models, sequential hydrolysis of two ATP molecules promotes the T-segment passage by induced conformational rearrangements [27, 28]. According to another model, the hydrolysis is required only for “restarting” the enzyme and trapping a new T-segment [29]. For example, in the presence of ADPNP, a non-hydrolyzable ATP analogue, topoisomerase is able to perform one act of T-segment passage, and then the enzyme remains in an inactive state with a closed N-gate [30]. According to recent single-molecule studies of DNA and DNA gyrase using magnetic tweezers, ATP hydrolysis is important both for accelerating T-segment passage and for “restarting” the enzyme [7]. An alternative explanation considers ATP binding and GHKL domain dimerization as a safeguard that is necessary to stabilize the two halves of the enzymatic complex and to prevent the formation of double-strand breaks during T-segment transfer due to accidental dissociation of the two enzyme halves [8].

The catalytic mechanism of type IIB topoisomerases is considered to be similar to that of type IIA topoisomerases (*Fig. 3B*) [31, 32, 33]. However, due to the absence of a C-gate, the T-segment immediately leaves the enzymatic complex after passing through the DNA-gate and the break in the G-segment [31]. In type IIB topoisomerases, the tyrosyl residues of WHDs are located on different secondary structure elements compared to the homologous domains of type IIA enzymes. When cleaving the G-segment of DNA, they generate two-nucleotide 5’-overhanging ends instead of the four-nucleotide overhangs characteristic of type IIA topoisomerases [34, 35]. G-segment cleavage was shown to depend on ATP binding for IIB enzymes. This is considered necessary for the stabilization of the complex and that of the temporary double-stranded break [8, 32].

The evolutionary relationships within type IIA and IIB topoisomerase groups and between these groups remain the subject of debate. Only a few evolutionary events can be reliably traced; for example, the duplication of a type IIA topoisomerase gene in the ancestor of bacteria, which led to the emergence of two enzymes with specific functions: DNA gyrase and Topo IV. Similarly, a duplication in the ancestor of vertebrates resulted in the emergence of Top2α and Top2β. Horizontal transfer of gyrase genes from different bacterial groups to Euryarchaeota and reverse transfer of Topo VI genes have also been described. Bacterial gyrase found in Archaeplastida is likely to be inherited from chloroplasts during establishing of primary endosymbiosis [10]. For more ancient events of topoisomerase evolution, there is no consensus.

## BACTERIAL TOPOISOMERASES

Free-living fast-growing bacteria, such as *Escherichia coli*, *Caulobacter crescentus*, and *Bacillus subtilis*, usually possess a wide spectrum of topoisomerases. This includes type I topoisomerases I and III, as well as type II, class IIA DNA gyrase and topoisomerase IV [4, 36, 37, 38]. Slow-growing bacteria (e.g., *Mycobacterium tuberculosis*) or symbiotic/parasitic bacteria with reduced genomes (e.g., *Helicobacter pylori*), in contrast, often have the minimum essential set of one type I (topoisomerase I) and one type II (DNA gyrase) enzymes [39, 40]. The genomes of several endosymbiotic bacteria, for example *Hodgkinia cicadicola *and *Tremblaya princeps,* lack topoisomerase II genes or, like *Carsonella rudii*, encode only one subunit [41, 42, 43]. These organisms have extremely reduced (139–160 kb) genomes.

DNA gyrase and topoisomerase IV are the targets for many antibiotics that, according to their mechanism of action, may be divided into two groups: poisons and catalytic inhibitors. Poisons stabilize an intermediate covalent complex of topoisomerase with the DNA G-segment. Accidental dissociation of enzyme subunits from such a complex (for example induced by the collision with the replisome or RNA polymerase) causes double-stranded DNA breaks and ultimately leads to cell death. Catalytic inhibitors do not cause DNA breaks, but they inhibit enzymatic activity, for example, by binding to the ATPase center of the GHKL domain and competing with ATP [44, 45].

Quinolone and fluoroquinolone drugs (ciprofloxacin, levofloxacin, etc.), which are often used in clinical practice, are topoisomerase poisons [44, 46]. Structural studies have shown that movement of divalent metal ions (most often magnesium) in the topoisomerase catalytic center is necessary for DNA cleavage and ligation. Gyrase poisons stabilize a metal ion in the position that promotes DNA cleavage, but not the sealing of the break [47, 48]. The latter fact explains the effects of the most prevalent gyrase mutations leading to antibiotic resistance. The conserved serine and glutamine residues of the WHD were found to coordinate water molecules and magnesium ions, which are necessary for the binding of fluoroquinolones [47]. Replacing at least one of these residues with a non-polar moiety leads to poison resistance [49].

Classical catalytic inhibitors are aminocoumarin compounds (novobiocin and coumermycin A1) that compete with ATP for the interaction with the ATPase center [44, 50]. Inhibition of gyrase activity leads to inhibition of replication and transcription and cell division arrest. Due to the low solubility of aminocoumarins and their toxicity to humans, aminocoumarin drugs are not currently used in clinical practice, but they found application in veterinary medicine [45].

The spread of antibiotic resistance necessitates a search for new antibacterial drugs; several new classes of topoisomerase inhibitors are currently in clinical trials [45, 51, 52].

**DNA gyrase**

**Fig. 4:**
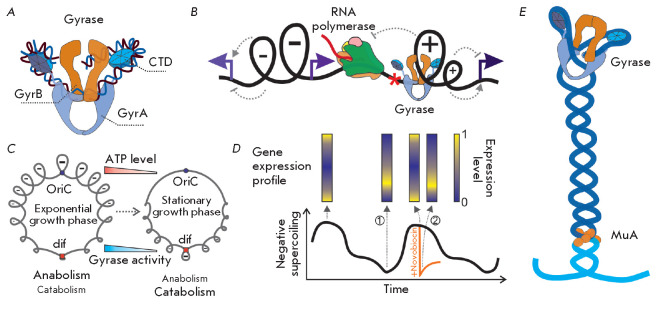
DNA gyrase and its function. (*A*) Structure of a DNA gyrase complex with DNA. (*B*) a twin-domain model illustrating positive supercoiling upstream of the elongating RNA-polymerase and negative supercoiling downstream [62]. Co-transcriptional positive, and negative, supercoiling moves along the DNA molecule and influences the initiation of transcription from adjacent promoters (indicated by arrows). Depending on the promoter, the effect can be either activating or inhibiting. DNA gyrase promotes transcription elongation through relaxation of positive supercoiling ahead of RNA polymerase. (*C*) Changes in genome supercoiling during *E. coli *culture transition from the exponential to stationary growth phase promote switching of the cell from a mainly anabolic to catabolic physiological state [63]. OriC – origin of replication, dif – site recognized by XerC/XerD recombinases. (*D*) Circadian oscillations of the *S. elongatus *genome supercoiling level (at the bottom) correlate with changes in the gene transcriptional profile (at the top). A sharp decrease in the genome supercoiling level (indicated by the orange arrow) in the presence of the DNA gyrase inhibitor novobiocin causes rapid change in the transcriptional profile (2), making it similar to the profile of bacteria in the physiologically relaxed genome state (1) [64]. (*E*) DNA gyrase is essential for the spatial organization of the Mu prophage and its transposition. The prophage DNA is shown in dark blue, and bacterial genome DNA is in blue

Bacterial DNA gyrases are conserved enzymes (*Fig. 4A*) sharing a unique ability to induce negative supercoiling using the energy of ATP hydrolysis, which was demonstrated in *in vitro *experiments for enzymes from *E. coli*, *B. subtilis*, *C. crescentus*, *M. tuberculosis*, and many other bacteria. In addition, DNA gyrases effectively relax positive supercoils and are capable of decatenating circular DNA molecules [39, 53, 54, 55, 56]. The* gyrA *and *gyrB *genes encoding the enzyme subunits are essential, and inhibitors that reduce gyrase activity significantly decrease cell viability [57, 58, 59, 60]. Gyrase inhibition induces a similar phenotype in different bacteria: elongated cells incapable of dividing [60, 61].

Gyrase maintains negative supercoiling of the genome, facilitating the initiation of transcription and replication. It also relaxes positive supercoils in front of elongating polymerases. Early ChIP-chip (immunoprecipitation of protein-bound DNA and its subsequent analysis on a chip to determine protein binding sites) experiments with *E. coli *revealed a positive correlation between gyrase binding and a gene’s transcription level [65]. Later, using the Topo-Seq method that enables highly accurate mapping of topoisomerase activity sites, catalytically active DNA gyrase from *E. coli* was directly shown to be located at the ends of active genes and in the regions downstream of transcription terminators [66]. Similarly, the results of ChIP-Seq (immunoprecipitation of protein-bound DNA and its subsequent sequencing to determine protein binding sites) experiments with *M. tuberculosis *gyrase indicate preferential binding of the enzyme to transcriptionally active regions [67]. In *C. crescentus*, suppression of the* gapR *gene expression inhibits initiation and elongation of replication and increases the sensitivity of cells to gyrase inhibitors. *In vitro *experiments have shown that the GapR protein preferentially binds to positively supercoiled DNA and interacts with the gyrase, increasing its ability to relax positive supercoils. Probably, GapR recruits the gyrase to the positive supercoils formed in front of the moving replication complex, fa- cilitating their relaxation and thus stimulating replication [55]. Single-molecule experiments have shown that in the absence of gyrase, transcription on topologically constrained DNA molecules quickly slows down and eventually stops due to the accumulation of positive supercoiling (*Fig. 4B*). The binding of gyrase to such molecules results in rapid restoration of the normal rate of transcription (transcriptional burst) [68].

In addition to its ability to relax positive supercoiling in front of elongating RNA polymerase, by introduction of negative supercoiling the gyrase can both activate and suppress transcription initiation [69]. Up to half of* E. coli *genes were found to respond to genome relaxation by changing their transcription level [70, 71]. Ontological analysis of *E. coli *genes sensitive to supercoiling revealed that the products of genes responding to relaxation of negative supercoils by increasing their transcription level are preferentially involved in catabolic reactions (for example, Krebs cycle enzymes). These genes are located closer to the terminus of replication. In contrast, genes that require negative genome supercoiling for initiation of their transcription are predominantly associated with anabolic processes (synthesis of amino acids and nucleotides) and are located closer to the region of replication origin [71, 72]. According to one model, during active growth of a *E. coli *culture, activity of DNA gyrase generates a negative supercoiling gradient in the genome, with the maximum and minimum levels being in the replication origin and the terminus regions, respectively. This leads to a predominant expression of the genes involved in the anabolic process, promoting cell growth and division. Depletion of nutrients in the stationary phase decreases the ATP concentration, which reduces DNA gyrase activity. This decreases the genome supercoiling level and, in combination with other factors, inverts the gradient of chromosome supercoiling, resulting in a predominant expression of the genes involved in catabolic processes [63]. It was hypothesized that *E. coli *uses supercoiling to globally modulate gene transcription upon starvation [72, 73, 74] (*Fig. 4C*).

Promoters of the *E. coli gyrA*, *gyrB*, and *topA *genes that encode gyrase and topoisomerase I subunits are highly sensitive to supercoiling. They contain supercoiling sensors: the *gyrA *and *gyrB *transcription is activated upon genome relaxation, while *topA *is better transcribed upon enhancement of negative supercoiling [75, 76]. This enables the mutually regulated synthesis of two topoisomerases with opposite activities, which provides a homeostat for the genome-wide supercoiling level [77, 78]. Similar mechanisms are operational in* S. coelicolor *and *C. crescentus *[58, 79].

The supercoiling level in *Salmonella typhimurium *is believed to regulate the transition from anaerobic metabolism to aerobic respiration [80]. In *H. pylori*, negative supercoiling is an important regulator of flagellar synthesis [81]. Circadian oscillations of DNA supercoiling in the cyanobacterium *Synechococcus elongatus* correlate with specific changes in gene transcription and relaxation of negative supercoiling by the addition of the DNA gyrase inhibitor novobiocin, leading to a rapid change in the gene transcription pattern, mimicking the changes observed during the circadian cycle (*Fig. 4D*) [64]. Overall, these data allow one to consider supercoiling as a global transcription factor and show that the structure of regulatory regions has evolved to allow specific responses to this factor [63, 69, 72].

A number of studies have indicated that gyrase and gyrase-induced negative supercoiling are involved in the spatial organization of bacterial genomes. For example, *in vivo *fluoroquinolone induces cleavage of* E. coli *genomic DNA by the gyrase into 50- to 100-kb fragments, which roughly corresponds to the length of supercoiled chromosome domains [82, 83, 84]. Activity of DNA gyrase at a high-affinity site located at the center of the bacteriophage Mu prophage was shown to cause a local increase in negative supercoiling, leading to plectonemic compaction of the chromosome region with the prophage. This brings prophage termini into proximity with each other and promotes their recombination by the MuA transposase [85, 86] (*Fig. 4E*). Similarly, excessive negative supercoiling accumulated in *E. coli *cells with a mutation in topoisomerase I is believed to lead to chromosome compaction [87]. As shown by Hi-C experiments (a method for determining the chromosome conformation) in *C. crescentus*, gyrase inhibition by novobiocin, on the contrary, makes the spatial structure of the chromosome more diffuse [88]. It should be noted that for the *E. coli *genome no significant associations between gyrase active sites and either the boundaries or locations of topologically associating domains (TADs) determined by Hi-C were found [66]. Further research is needed to elucidate the role of supercoiling in the regulation of the spatial organization of prokaryotic genomes.

**Topoisomerase IV**

**Fig. 5:**
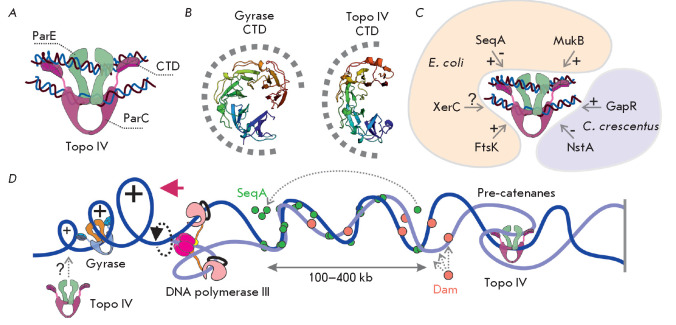
Topoisomerase IV and its function. (*A*) Structure of the Topo IV complex with DNA. (*B*) Comparison of the GyrA CTD (PDB ID: **1zi0**) and Topo IV ParC CTD (PDB ID: **1zvt**) structures. A putative position of DNA is shown as a dashed line. (*C*) Proteins interacting with Topo IV. The effect of each protein on Topo IV activity is depicted as “+” (activation), “–” (inhibition), or “?” (interaction is not confirmed). (*D*) Topological effects associated with DNA replication. Positive supercoils formed in front of the moving replisome are relaxed by DNA gyrase and, presumably, Topo IV. Accumulation of DNA supercoiling leads to replisome rotation, thereby producing DNA pre-catenanes. In *E. coli*, the SeqA protein binds to the hemimethylated GATC sites of newly replicated DNA molecules. Dam methylates GATC sites and displaces SeqA; so, the SeqA concentration gradient extends 100–400 kb over the replisome and moves together with it. Topo IV cannot interact with SeqA-bound DNA regions, which explains the temporary cohesion of daughter chromosomes during replication in *E. coli*; however, when all GATC sites are methylated and SeqA is no longer associated with DNA, topoisomerase removes pre-catenanes, enabling daughter chromosome separation [110]

*In vitro *experiments have demonstrated that despite their structural similarity topoisomerases IV (Topo IV) and gyrases have different spectra of activity. Topo IV is able to effectively relax positive supercoils. Negative supercoils are relaxed at a much slower rate. Unlike the gyrase, Topo IV cannot introduce excessive negative supercoiling [55, 56, 89]. At the same time, Topo IV is an efficient decatenase that separates interlinked circular DNA molecules much better than gyrase [90-94]. Accordingly, Topo IV, but not gyrase, is capable of resolving knotted DNA molecules *in vivo* [95]. It is hypothesized that these differences are related to the structures of CTD domains in the GyrA subunit of gyrase and in the homologous ParC subunit of Topo IV (*Fig. 5B*). The gyrase CTD enables wrapping of DNA around the enzyme, such that DNA located* in cis *and close to the G-segment of DNA serves as a T-segment, which allows for the introduction of negative supercoils in one DNA molecule [7, 96]. The Topo IV CTD does not bend the G-segment; instead it traps as a T-segment remote DNA sites or *in trans* DNA molecules. Since the T-segment must be perpendicular to the enzyme-bound G-segment, catenanes are effectively recognized and resolved [89, 93, 97] (*Fig. 4A*, *Fig. 5A*).

Like gyrase, Topo IV is necessary for bacterial division. Mutations in the *parC *and *parE *topoisomerase subunit genes or inhibition of the enzyme activity by drugs causes the development of the so-called *par* phenotype in different bacteria. The *par *phenotype is characterized by elongated cells that are not capable of division and contain an increased amount of unsegregated DNA [36, 98, 99, 100, 101]. However, the lack of Topo IV activity does not interfere with *E. coli *chromosome replication and its termination [99, 100]. The biochemical properties of the enzyme suggest that the main function of Topo IV in the cell is to resolve pre-catenanes during replication (intersections between sister DNA molecules arising from replisome rotation) and to separate catenanes of circular molecules upon the completion of replication [100, 102] 2] (*Fig. 5D*). According to this hypothesis, Topo IV is not essential for* Streptomyces *with linear chromosomes but is important for the maintenance of circular plasmids [38]. Yet,* E. coli *cells with artificial linear chromosomes exhibit* par *phenotype upon Topo IV inactivation. This may be an indication of the importance of the early removal of pre-catenanes and knots along the entire length of the replicating chromosome [103]. An increase in the Topo IV expression level leads to accelerated DNA segregation during the division of *E. coli *cells [100].

The ability of Topo IV to relax positive supercoils [56, 89] suggests that it may cooperate with the DNA gyrase in the removal of positive supercoils formed during transcription and replication [55, 106] (*Fig. 5D*). For example, treatment of *E. coli *cells with the RNA polymerase inhibitor rifampicin was found to reduce both the gyrase and Topo IV activities, at least in some regions of the genome [83, 107]. Interestingly, an increase in the copy number of the *parC *and *parE *genes is a common suppressor mutation associated with deletion of the topoisomerase I gene in *E. coli *and *B. subtilis*. In this case, Topo IV is believed to compensate for the loss of topoisomerase I and perform its function by removing negative supercoiling [37, 98, 108].

Topo IV interacts with a number of proteins that have completely different functions and structures, but are involved in the organization and separation of replicated chromosomes. In *E. coli*, these are the SeqA protein that binds to the hemimethylated GATC sites behind the moving replisome [109, 110], the MukBEF cohesin [111, 112], the DNA translocase FtsK [113], and, probably, the XerC recombinase [107, 114] (*Fig. 5C*). *C. crescentus *Topo IV interacts with GapR and NstA. These proteins have opposite effects on Topo IV – GapR stimulates enzyme activity, while NstA suppresses it [55, 115]. *In vivo*, Topo IV and the *E. coli* cohesin complex MukBEF form clusters consisting of ~15 topoisomerase molecules and ~10 cohesin molecules [116, 117]. These clusters colocalize with replication origins, determine their position in the cell, and are necessary for segregation of the origins of daughter chromosomes during division [116, 118, 119].* C. crescentus *Topo IV is also required for the correct movement of one of the origins to the opposite cell pole [101].

**Topoisomerase NM**

A unique type II topoisomerase, called TopoNM, was discovered in *M. smegmatis *[120]. It consists of two subunits (TopoN and TopoM), homologous to the ParE/GyrB and ParC/GyrA subunits of topoisomerase IV and gyrase, respectively. According to a phylogenetic analysis of amino acid sequences, TopoNM is distant from all known type IIA topoisomerases, which indicates early divergence of enzyme genes [120]. The significant divergence from other topoisomerases II and the absence of TopoNM in other, even related, bacteria may indicate the viral origin of the enzyme. TopoNM has reduced sensitivity to fluoroquinolones and coumarins. The enzyme relaxes positive and negative supercoils and decatenates circular DNA molecules, which is typical of type II topoisomerases. A unique property of TopoNM is the ability to introduce positive supercoils into relaxed plasmids [120]. Besides TopoNM, only reverse gyrase – a type I topoisomerase – is capable of introducing positive supercoils using the energy of ATP hydrolysis [121]. Neither the mechanism of positive supercoiling by TopoNM nor the functions of this enzyme are known.

An unusual system for protection against mobile genetic elements was found in *M. smegmatis*. It consists of genes encoding a cohesin-like complex that prevents effective transformation of bacteria with plasmids [122, 123]. TopoNM may be part of this defense system, in the way some bacterial topoisomerases interact with cohesins [111, 112, 124].

## ARCHAEAL TOPOISOMERASES

Members of the Archaea domain usually harbour type IIB topoisomerases (Topo VI). Some archaea from the Euryarchaeota phylum have lost their Topo IV genes but independently acquired, through horizontal gene transfer, DNA gyrase genes from different bacterial groups [11]. Hyperthermophilic archaea encode reverse gyrases as an adaptation to high temperatures, since this enzyme is believed to be essential for maintaining DNA duplex stability at high temperatures and is involved in DNA repair [125, 126, 127].

**Topoisomerase VI**

Topoisomerase VI (Topo VI) was first found in the hyperthermophilic archaeon *Sulfolobus shibatae *[128] and, later, in most other archaea, except for some members of the Thermoplasmatales group in which it is replaced by the DNA gyrase [11]. *In vitro*, Topo VI can relax both positive and negative supercoils and exhibits decatenation activity [32, 129]. Similarity between the amino acid sequences of IIA and IIB topoisomerases is rather low. Additionally, the catalytic tyrosine residues of WHDs are located on non-homologous secondary structure elements in the two groups [32, 33, 130] (*Fig. 2*). Despite these, the catalytic mechanism of Topo VI is supposed to be similar to that of type IIA topoisomerases, a conclusion based on biochemical and structural analyses (*Fig. 3B*).

The physiological role of Topo VI has not been established. The activity of the enzyme demonstrated *in vitro* and the fact that Topo VI can be replaced with DNA gyrase indicate that the topoisomerase may be involved in the decatenation of replicated chromosomes and in the relaxation of supercoils formed during transcription and replication [129]. The expression level of Topo VI in *S. islandicus *was found to increase 7 h after one elevates the cultivation temperature above its optimal level. Probably, Topo VI compensates for an increase in reverse gyrase activity under these conditions [131].

**DNA gyrase**

Gyrase genes have been found in members of several Euryarchaeota groups [11]. Like bacterial gyrase, the archaeal enzyme is sensitive to coumarins and quinolones [132, 133, 134]. *In vitro *experiments have shown that *Thermoplasma acidophilum *gyrase has a typical spectrum of activities: it relaxes positive supercoils, introduces negative supercoils, and decatenates circular DNA molecules [134]. Inhibition of gyrase activity by the addition of novobiocin to* Halobacterium halobium *cells leads to the inhibition of DNA replication and a significant decrease in the levels of transcription and translation [132]. Thus, the archaeal gyrases are believed to perform functions typical of bacterial homologues: relaxation of positive supercoils formed during transcription and replication, as well as decatenation of linked DNA molecules during cell division.

## EUKARYOTIC TOPOISOMERASES

Homodimeric topoisomerase IIA (Top2) is common to all known eukaryotes. It is encoded by one *Top2 *gene in most species; vertebrates, however, have two paralogous genes, *Top2α *and *Top2β *[10]. Archaeplastida and eukaryotes related to them via secondary endosymbiosis of plastids (Apicomplexa, etc.) contain DNA gyrase genes. The enzyme is of bacterial origin and is encoded by nuclear genes that had been transferred from the chloroplast genome after the establishment of endosymbiosis [11, 135]. The ubiquitous eukaryotic proteins involved in a complex required for generating DNA breaks during meiotic recombination are homologous to Topo VI from Archaea: Spo11 and Rec102/ Rec6/MEI-P22 are homologues of the A and B subunits respectively [128, 136, 137]. These proteins are not considered topoisomerases, and we will not discuss them in detail. However, a full-length heterotetrameric Topo VI possessing typical enzymatic activities is found in plants, making it another distinctive feature of Archaeplastida [138].

Most agents used in cancer chemotherapy are topoisomerase poisons, with etoposide being the most common [139-145]. They induce double-strand breaks (DSBs), thus causing apoptosis [146-151]. The selectivity of these drugs is determined by the neoplastic features of tumor cells: they actively proliferate and have an increased topoisomerase expression level [152]. Severe side effects caused by DNA damage in normal cells, especially actively proliferating, remain a crucial issue in chemotherapy [153, 154]. Top2-mediated DSBs can lead to chromosomal translocations and induce secondary malignancies [155]. For example, etoposide therapy often leads to secondary leukemia [156, 157, 158]. The oncogenic effects often arise due to the inhibition of Top2β that is actively expressed in most tissues and is associated with promoter regions [159-163]. A possible solution to this problem may be searching for and using inhibitors targeting Top2α selectively.

Catalytic inhibitors of Top2 (merbarone, suramin, bis-dioxypiperazine derivative ICRF-187) have not been used broadly in clinical practice as antineoplastic drugs [164]. However, some of them are used as cardioprotectors, simultaneously with oncotherapy involving Top2 poisons [165, 166]. According to one of the existing hypotheses, the protective properties of inhibitors are associated with a decrease in the number of DNA-Top2 covalent complexes and, accordingly, DNA breaks due to inhibition of Top2 activity [167, 168].

**Top2**

Eukaryotic Top2 is a classic type IIA topoisomerase. It relaxes positive and negative supercoils and decatenates DNA molecules [169-172]. Top2 inactivation impairs chromatin condensation, leads to changes in chromosome morphology, chromosomal rearrangements, and abnormalities of embryogenesis and nervous system development in vertebrates [170, 173-180].

Eukaryotic Top2 primarily has a nuclear localization but is also present in the mitochondria of mammalian cells [181]. An increased expression level of the *Top2* gene (*Top2α *in vertebrates) is common to actively proliferating tissues, since the enzyme is essential to chromosome condensation and separation during mitosis [182, 183]. The level of *Top2β *gene expression is less dependent on the tissue type [184].

The Top2 CTD is the least conserved Top2 region. The CTD undergoes post-translational modifications, most prominently, phosphorylation, which changes in a cell cycle-dependent manner. The divergence between Top2α and Top2β CTDs determines the functional differences between the paralogs and their regulation [185]. By studying the properties of chimeric enzymes (Top2α with the CTD of Top2β and *vice versa*) it was demonstrated that Top2α CTD (CTDα) attracts topoisomerase to chromosomes during mitosis and that a topoisomerase with CTDα is required for cell proliferation [186]. In contrast, CTDβ was shown to decrease the affinity between topoisomerase it is attached to for DNA and reduce the efficiency of catalysis [187, 188].

**Fig. 6:**
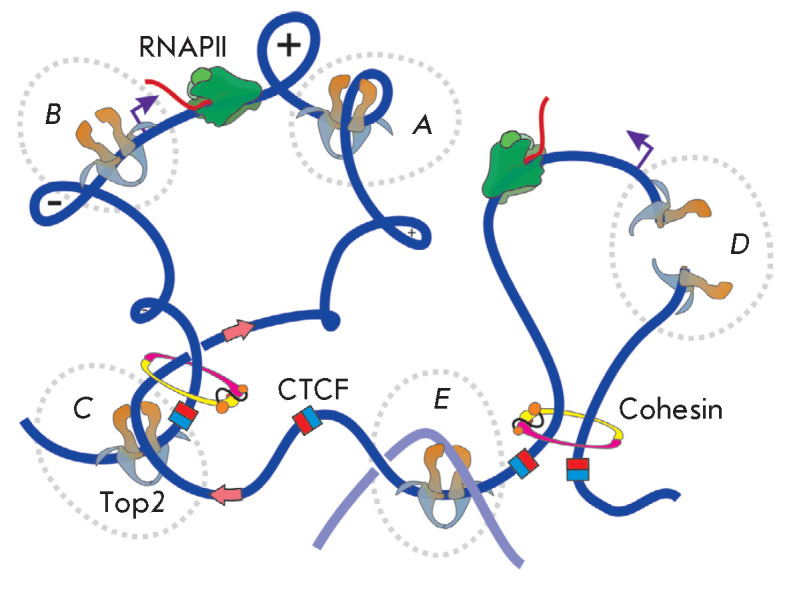
Function of eukaryotic Top2. (*A*) Relaxation of supercoils during transcription. Promoters are depicted by purple arrows. (*B*) Top2 is involved in transcription initiation. (*C*) Top2, CTCF, and cohesin are colocalized at the TAD boundaries. Pink arrows display the direction of loop extrusion, mediated by CTCF and cohesin. Red-blue squares depict CTCF binding sites. Top2 facilitates cohesin- mediated DNA translocation through the relaxation of topological stress. (*D*) Top2-mediated introduction of DNA double-strand breaks in the promoter region induces transcription. (*E*) Decatenation of daughter chromosomes

Top2 is required during the transcription of highly active and, especially, long genes. It relaxes positive supercoils in front of the elongating RNA polymerase (*Fig. 6A*) [189-193]. Moreover, Top2 recruits RNA polymerase II to gene promoters [194, 195] and plays an important role in the transcription initiation of some inducible yeast genes (*Fig. 6B*) [196]. Induction of genes regulated by nuclear receptors (androgens, estrogens, glucocorticoids) is associated with the promoter- mediated assembly of a complex comprising chromatin-remodeling factors (BRG1), components of the DSB repair system (PARP1, Ku70), and Top2β [197-200]. In response to hormones, Top2β, which is part of this complex, introduces a double-strand break in DNA, efficiently relaxing supercoils during transcription (*Fig. 6D*). Similar data on the activating effect of Top2β-induced breaks were obtained for several genes in NMDA-stimulated neurons [201].

Recent studies have shown that, with rare exceptions, eukaryotic genomes are organized into topologically associating domains (TADs) [202, 203, 204]. Architectural proteins, particularly CTCF and cohesin, are associated with TAD boundaries [205, 206]. Colocalization of these proteins and Top2β at the boundaries of TADs was established using the ChIP-Seq and ChIP-exo approaches (the later method has enhanced precision because of exonuclease treatment of DNA– protein complexes) (*Fig. 6C*) [207]. In addition, mapping of Top2–DNA cleavage sites stabilized by etoposide has demonstrated that they are predominantly located near the CTCF binding sites [208, 209, 210, 211]. Presumably, TADs are composed of loops formed by extrusion due to the activity of cohesin and CTCF [212, 213, 214]. Top2 is supposed to play an important role in the functioning of chromatin loops and is necessary in order to relieve the topological stress at TAD boundaries (*Fig. 6C*). TAD compactization, according to some models, may be maintained due to the negative DNA supercoiling that can be considered a universal factor that spatially organizes both prokaryotic and eukaryotic genomes [215].

Top2 was also found to interact with the ATP-dependent chromatin-remodeling complexes [171, 216, 217, 218] that perform nucleosome assembly and movement along the DNA, and to replace canonical histones with histone variants, thus maintaining a tissue-specific chromatin structure [219, 220, 221]. The interaction between Top2 and remodeling complexes affect the catalytic properties of topoisomerase and its ability to bind DNA [171, 222], which is probably required for structural rearrangements within chromatin. The chromatin remodelers might be responsible for recruiting topoisomerases and CTCF to the TAD boundaries [223]. To date, the interplay between the chromatin architecture and Top2 activity has remained insufficiently explored and requires further investigation.

**DNA gyrase**

Eukaryotic gyrase, similarly to a bacterial enzyme, is capable of introducing negative supercoils *in vitro* and is sensitive to coumarins and quinolones [224, 225, 226]. Plant gyrase is able to complement a mutated enzyme in *E. coli *[225, 227, 228].

**Fig. 7:**
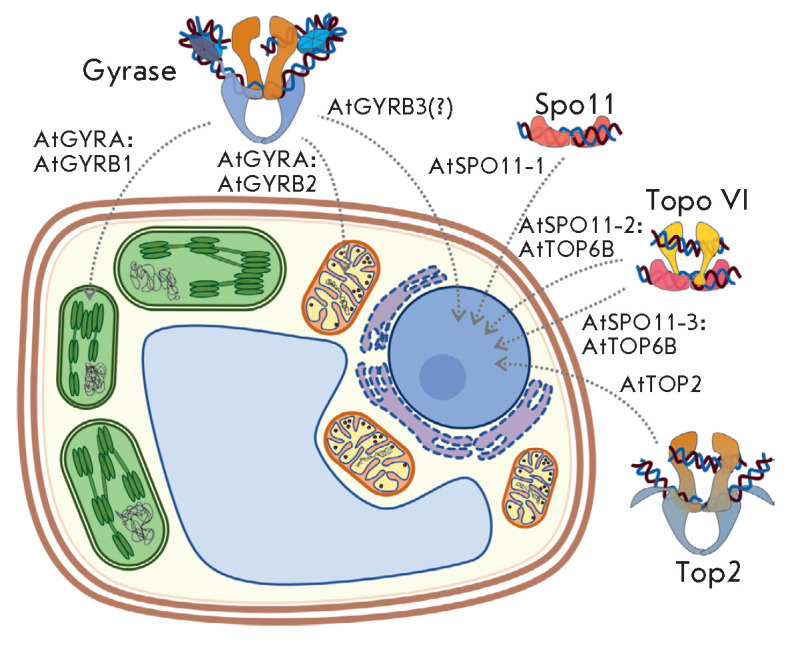
Cellular localization of type II topoisomerases and homologues proteins in *A. thaliana*

The nuclear genome of *Arabidopsis thaliana *contains one gene encoding the GYRA subunit and three paralogous genes encoding the GYRB subunit of gyrase [225]. It was shown that AtGYRA interacts with AtGYRB1 and AtGYRB2, forming complexes capable of introducing negative supercoils. In contrast, AtGYRA does not interact with AtGYRB3 [228, 229]. B-subunits contain signal peptides that are responsible for the localization of AtGYRB1 and AtGYRB2 in chloroplasts and mitochondria, respectively. Therefore, it is believed that the AtGYRA:AtGYRB1 complex functions in chloroplasts, and that the AtGYRA:AtGYRB2 complex functions in mitochondria. The AtGYRB3 subunit lacks a canonical signal peptide, but it is believed to localize in the nucleus [225, 228] (*Fig. 7*). *N. benthamiana *has one *GYRA *gene and two *GYRB *genes, with the GYRA and GYRB1 subunits being localized in chloroplasts and mitochondria [227]. Similar results were obtained for the GYRA subunit of *Pisum sativum *[230].

In plants, gyrase inhibition primarily affects chloroplasts and mitochondria. For example, treatment of* Chlamydomonas reinhardtii *algae with enzyme inhibitors (nalidixic acid, novobiocin) leads to transcription alteration in chloroplasts [224]. The addition of the nalidixic acid to *Nicotiana tabacum *cell cultures suppresses DNA synthesis in plastids [231]. Gyrase inhibitors reduce the number of chloroplasts and mitochondria, change the structure of chloroplasts, and, probably, disrupt their division [229]. Cultivation of plants on media supplemented with gyrase inhibitors or treatment of *A. thaliana *plants with these compounds retards their growth and induces etiolation, which ultimately leads to plant death [225, 229]. Similar results were obtained by suppression of gyrase expression in *N. benthamiana* plants by virus-induced gene silencing (VIGS), and in *A. thaliana *by RNA interference [227, 229]. Such data suggest that the enzyme in plants probably retains its characteristic role in double-membrane organelles and is necessary for the segregation of DNA, division, and transcription.

The role of the AtGYRB3 subunit remains unknown. This polypeptide lacks some amino acid motifs in its ATPase domain, which are conserved in type II topoisomerases. At the same time, it contains a histone-binding SANT domain not found in other topoisomerases [228, 232]. Analysis of *AtGYRB3 *gene expression in* A. thaliana *revealed no correlation with the expression of other gyrase genes: the highest expression level of* AtGYRB3 *was found in the stamens and pollen, while expression of the other subunits was most active in the seeds and shoot apical meristem. We hypothesize that the AtGYRB3 protein could be involved in meiosis, where it assists Topo VI or SPO11.

Bimolecular fluorescence complementation (BiFC) and co-immunoprecipitation experiments revealed some interaction between RNase H1 (AtRNH1C), which removes RNA from RNA–DNA heteroduplexes (R-loops) formed during transcription, and the AtGYRA subunit in *A. thaliana *chloroplasts [233]. The interaction between enzymes was thought to promote replication fork progression through R-loops that often form in actively transcribed regions of the chloroplast genome; e.g., in rRNA genes.

**Topoisomerase VI**

Among eukaryotes, the full-length heterotetrameric Topo VI is found only in *Archaeplastida *[138]. Similar to the case of gyrase, plants contain several paralogous genes encoding Topo VI subunits. *A. thaliana *has one B subunit gene (*AtTOP6B*) and three A subunit genes* AtSPO11-1,2,3*, while *Oryza sativa *has five paralogous genes of A-subunits and one gene of B-subunits [138, 234, 235]. Plant Topo VI subunits are localized in the nucleus, which had been predicted bioinformatically and was confirmed by microscopy [234, 236, 237].

Two-hybrid screening and co-immunoprecipitation experiments revealed that not all A-subunits form a complex with the B-subunit: *in A. thaliana*, AtTOP6B interacts with AtSPO11-2 and AtSPO11-3, but not with AtSPO11-1; in *O. sativa*, OsTOP6B interacts with OsSPO11-2, OsSPO11-3, and OsSPO11-4, but not with OsSPO11-1 and OsSPO11-5 [138, 234, 235]. The A subunits, which do not interact with the B subunit, likely function as Spo11 proteins in other eukaryotes. For example, AtSPO11-1 and OsSPO11-1 are required for meiotic recombination [238, 239]. Although OsSPO11-4 interacts with OsTOP6B, it is also required for meiosis in pollen grains; therefore, participation in this process may be one of the functions of plant Topo VI [235].

Mutations in or suppressed expression of the genes of Topo VI subunits that form full-length topoisomerase and are not involved in meiotic recombination cause a dwarf phenotype in plants and a decrease in cell size. These plants lack trichomes and root hairs [237, 240, 241]. Mutants were shown to have impaired endoreduplication – somatic cell polyploidization that normally occurs in plant cells [237, 241]. For efficient functioning, Topo VI forms a complex with the MID, RHL1, and BIN4 proteins (interestingly, RHL1 and BIN4 are distantly similar to the Top2α CTD of vertebrates) [237, 242, 243]. This complex is believed to participate in the regulation of the endoreduplication cycles and, probably, decatenation of chromosomes in cells with high ploidy [236, 237, 242, 243].

Overexpression of the Topo VI components of several plants in *A. thaliana *increases cell ploidy and significantly stimulates the resistance of organisms to stress conditions, such as increased salt content or drought, and reduces the sensitivity of plants to stress hormone abscisic acid [234, 241]. Overexpression of topoisomerase genes changes the levels of many transcripts. For example, it leads to the activation of stress-response cascades [234]. Topo VI was found to be also involved in plant response to oxidative stress through binding to the promoters of some genes [244]. The mechanism by which Topo VI affects transcription – relaxation of supercoils, introduction of breaks in DNA (like Top2β), or chromatin remodeling – remains unknown. In addition, it is not clear how endoreduplication and response to stress, both processes that involve Topo VI, are related.

## TOPOISOMERASES OF VIRUSES AND MOBILE GENETIC ELEMENTS

**Top2-like topoisomerases**

Viruses with large double-stranded DNA genomes (e.g., T4-like viruses and nucleo-cytoplasmic large DNA viruses (NCLDV)) encode their own Top2-like enzymes [11]. NCLDV topoisomerases (eukaryotic viruses) are a sister group of Top2 of their hosts. The phylogenetic position of bacteriophage T4 topoisomerases is less certain; their amino acid sequences are equally distant from those of bacterial and eukaryotic type IIA enzymes [11]. However, the structure and activity of these virus topoisomerases are conserved: the bacteriophage T4 enzyme, which is encoded by three genes, relaxes supercoils, decatenates circular DNA, and is sensitive to some Top2 inhibitors [245, 246]. Topoisomerases are believed to be necessary for the removal of positive supercoils that arise during the replication of the viral genome [247, 248].

**DNA gyrase**

DNA gyrase genes have been predicted in the genome of the giant bacteriophage AR9 and several related viruses from the Myoviridae group [249]. The functions and role of this enzyme are unknown.

**Topoisomerase VIII**

Genes of topoisomerases with predicted domains similar to the Topo VI domains are found in some archaeal and bacterial plasmids, as well as in integrated mobile genetic elements. The topoisomerases encoded by these genes are allocated into a separate group of type IIB topoisomerases and are referred to as “Topo VIII” [250, 251]. Several Topo VIII were shown to relax supercoiled plasmids and decatenate circular DNA molecules* in vitro *[250]. Recently, a new group of proteins homologous to the A-subunit of Topo VIII was identified; they are called Mini-A because of their relatively small size (*Fig. 2*) [251]. The function of these topoisomerases is unknown. Probably, they help to maintain plasmids and promote their propagation in host cells.

## CONCLUSION

Topoisomerases resolve topological problems that arise from DNA helicity. These enzymes are rather abundant and are required for fundamental cellular processes. According to one hypothesis, topoisomerases arose and spent the early stages of their evolution in viruses where they formed all known groups at or before the time when the last universal common ancestor (LUCA) existed. During the division of cellular organisms into modern domains, viruses spread, transferred, and mixed topoisomerase genes [11, 250]. It is likely that the variety of topoisomerases is only the tip of the iceberg, and that further exploration of “viral dark matter” could lead to the discovery of new types and classes of enzymes with unusual properties.

## Abbreviations

LUCA: last universal common ancestor
CTD: C-terminal domain
TAD: topologically associating domain
kb: kilobase
